# Is it the end of the road for dental amalgam? A critical review

**DOI:** 10.4103/0972-0707.45247

**Published:** 2008

**Authors:** Arvind Shenoy

**Affiliations:** Department of Conservative Dentistry, Bapuji Dental College, Davangere, Karnataka, India

**Keywords:** Composite resins, dental amalgam, failure, longevity

## Abstract

The longevity of dental restorations is dependent on many factors, including those related to materials, the dentist, and the patient. Dental amalgams have successfully served the profession for over a century. The main reasons for restoration failure are secondary caries, fracture of the bulk of the restoration or of the tooth, and marginal deficiencies and wear. The importance of direct-placement, aesthetic, tooth-colored restorative materials is still increasing. Amalgam restorations are being replaced because of alleged adverse health effects and inferior aesthetic appearance. All alternative restorative materials and procedures, however, have certain limitations. This article will attempt to critically analyse both amalgams and resin based composites, through an evaluation of scientific literature.

## INTRODUCTION

According to the American Dental Association (ADA), more than 100 million silver-amalgam fillings are placed in American mouths each year. A cursory search through the Internet throws up a horde of articles, speculative, informative, and even downright absurd. The fact of the matter remains that silver amalgam is still the most widely used restorative material in the developing world. In recent years, however, its usage has decreased dramatically and we are at that curious juncture, where people are beginning to ask, ‘Is it the beginning of the end for silver amalgam?’

I remember reading a news item regarding the ban being imposed on dental amalgam in Norway. The Norwegian Government (Ministry of Environment) passed this legislation on December 14, 2007. It aims to prohibit the production, importation, exportation, sale, and use of substances that contain mercury.

In India, the use of amalgam has been decreasing over the years, not as much because of public perception on mercury toxicity or regulatory issues but due to the increased demand for esthetic restoratives.

There seems to be a curious reluctance to accept composite resins as a viable alternative to amalgams, and, more often than not, this reluctance can be attributed to anecdotal rather than evidence based data. This leads to a situation where most students who graduate are woefully inadequate, with little or no clinical skills in placing composite restorations. Posterior composite resin restorations are an established feature of contemporary dental practice and all new dental graduates should be competent in providing such treatment for their patients.

As an academician, I find that most dental institutions passionately hold on to amalgam as the material of choice for undergraduates. Surveys of educational curricula in this area, in the United Kingdom and Ireland, as well as North America, have demonstrated variations both within and between dental schools.[1] At the British Association of Teachers of Conservative Dentistry Annual Conference held in Birmingham in September 2005, a session was devoted to the development of guidelines for dental schools on teaching posterior composite resin restorations to dental undergraduates. The theme of the conference concerned the teaching implications for changing from amalgam to composite. Perhaps the time is ripe for such a discussion in India as well.

## DENTAL AMALGAM

Over its long clinical history, dental amalgam has evolved, and current clinical restorations represent an effective low cost dental restorative treatment, with potential clinical lifetimes in excess of several decades, under appropriate conditions. Modern amalgams encompass a number of broad classes of materials, with variations in phase content, making them the most metallurgically complex biomaterials.

Amalgam restorations undergo a variety of solid-state and corrosion reactions after clinical placement. This presents complications in the understanding of clinical properties and biological characteristics. Dental amalgam classes contain a number of common phases and undergo similar solid-state and corrosion reactions, some of which offer the potential for the release of mercury (Hg). The major phases which release Hg as a result of these processes include the *γ* Ag-Hg matrix phase and the γ2 phase, which occurs mainly in traditional low-copper amalgam formulations. Most of the Hg which is released appears to react with residual alloy particles.[2]

## DURABILITY OF AMALGAM RESTORATIONS

**Figure 1:**
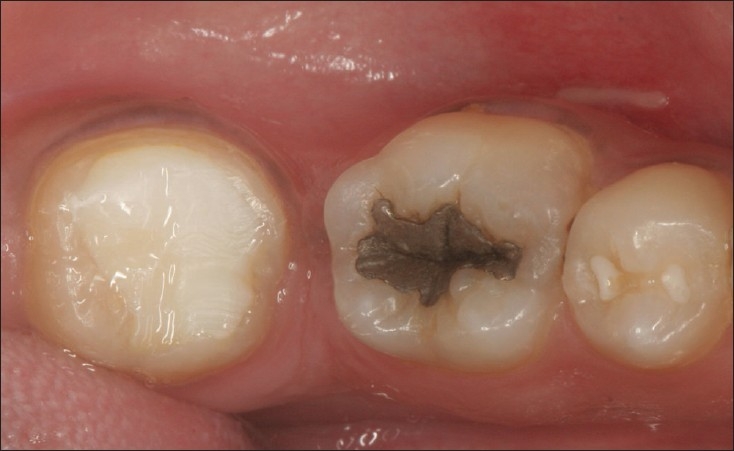
15 year old amalgam restoration in 36 which shows some evidence of corrosion and extrusion but continues to function efficiently

**Figure 2:**
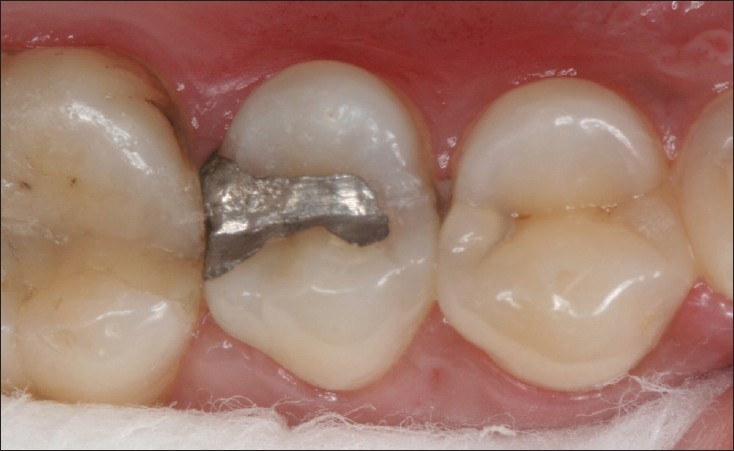
35 disto-occlusal restoration at 10 years

**Figure 3:**
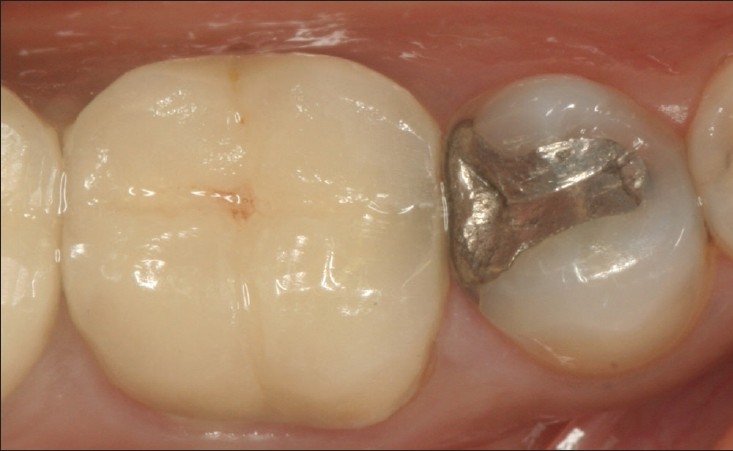
Well polished amalgam restoration in perfect shape after 10 years of service. No evidence of corrosion, marginal discrepancy and secondary caries

Recent research shows that amalgam restorations last longer than was previously thought [Figures 1-3]. The older generation of low-copper amalgams (before 1963) did have a limited life span, because they contained the gamma 2 phase that caused progressive weakening of the amalgam through corrosion.[3] Several clinical studies have demonstrated that high-copper amalgams can provide satisfactory performance for more than 12 years [Figure 3].[4–8] This appears to be true even for large restorations that replace cusps.[9] In addition, high-copper amalgams do not appear to require polishing after placement, to increase their longevity, as was recommended for low copper amalgams.[10]

Plasmans *et al*.[11] evaluated the long term survival of large multi-surface restorations and found that extension of extensive amalgam restoration (i.e., the number of cusps involved in the restoration) had no influence on the survival rate, which is in accordance with the results of a retrospective study by Robbins and Summitt, who found a 50% survival rate of 11.5 years.[12]

The reason for the satisfactory functioning of the extensive amalgam restorations over a long period of time results from the prevention of the most important traditional mechanical failure of amalgam restorations. These include marginal fracture, bulk fracture and tooth fracture.[13,14] With careful evaluation of cusp strength and the reduction of weak cusps, these types of clinical failures can generally be prevented. The zinc and copper content of the alloy has been found to have a strong impact on the survival rates of amalgam restorations, since it influences the corrosion resistance of the amalgam. High-copper amalgams have higher survival rates than conventional amalgams.[13]

Manhart *et al*.[15] completed a review on the longevity of restorations in stress-bearing posterior cavities and assessed the possible reasons for clinical failure. Since 1990, dental literature was predominantly reviewed for longitudinal, controlled clinical studies and retrospective cross-sectional studies of posterior restorations. The mean (SD) annual failure rate in posterior stress-bearing cavities was 3.0% (1.9) for amalgam restorations. The main problems limiting the survival of amalgam restorations were reported to be secondary caries, a high incidence of bulk and tooth fracture, cervical overhang and marginal ditching.

Letzel[16] investigated survival and modes of failure of amalgam restorations retrospectively. The leading mode of failure was bulk fracture (4.6%), followed by tooth fracture (1.9%), and marginal ridge fracture (1.3%). For other reasons, 0.8% of the restorations failed.

## TOXICITY OF AMALGAMS

The debate over the safety and efficacy of amalgam has raged since time immemorial. In recent times, it has reached such a feverish pitch that it seems to drown out all sounds of reason.

Amalgam has served the dental profession for more than 150 years. Incidents of true allergy to mercury have been rare (only 41 cases have been reported since 1905), and attempts to link its usage with such diseases as multiple sclerosis and Alzheimer's have not been scientifically proven, although there may be some association between amalgam restorations and oral lichen-oid lesions.

Marshall,[2] in his review on Dental Amalgam, summed it up appropriately: ‘If some reported values of Hg release are extrapolated to clinical lifetimes, the entire restoration could lose its Hg in a short time. For example, a 500-mg amalgam restoration contains approximately 200-250 mg of Hg, and the entire quantity of Hg would be lost in 10,000 days if the Hg was released at the rate of 25 ug/day. This estimate of release is of the order of magnitude reported in some studies of vapor release.’

As recently as May 2005, the ADA endorsed amalgam as being safe for pregnant women. Still, the anti-amalgamists persist in their efforts to discredit the dental profession. A discussion on this issue is beyond the scope of this article but a good starting point would be Hyson's[17] treatise on the history of amalgam, in which he has discussed the issue in detail.

## COMPOSITE RESINS

‘Durability is a major problem with posterior composites. The typical life-span of posterior composites is from three to 10 years, with large fillings usually fewer than five years. Polymerization shrinkage and inadequate adhesion to cavity walls are remaining problems. Some pulp irritation can occur if deep restorations are not placed over a protective film.’ Bowen.[18]

A lot has changed since Bowen made this statement in 1992. As a sign of the times, in 1999, around 86 million composite restorations were placed in the United States, as against 71 million amalgam restorations. The reasons were improvements in com-posite materials and techniques, and public demand for more esthetic, tooth-colored restorations.[17]

**Figure 4:**
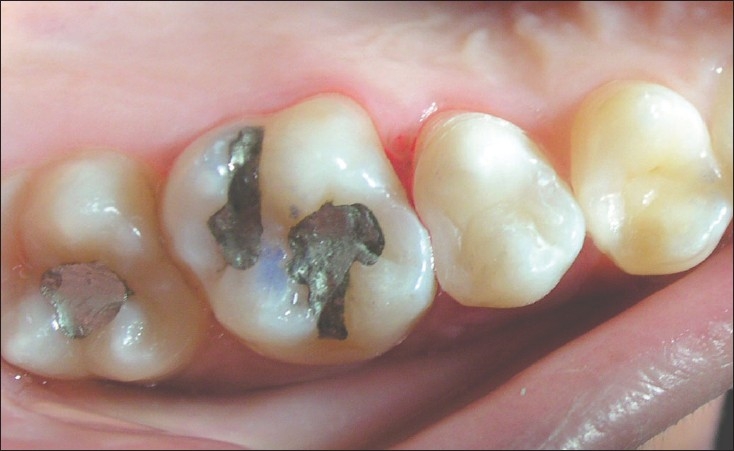
Disto occlusal composite restoration in 15

**Figure 5:**
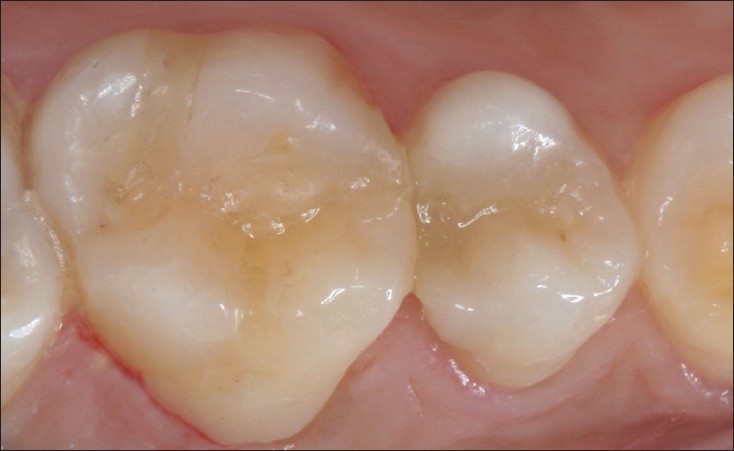
15 and 16 restorations at 3 years in perfect function, showing minimal discoluration

**Figure 6:**
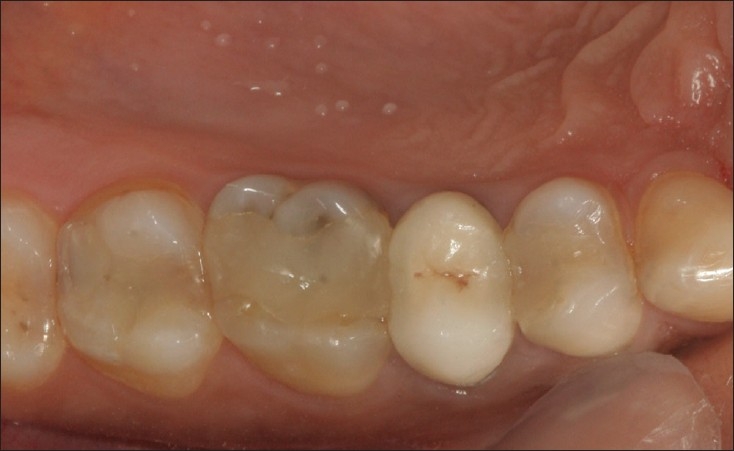
Multiple large composite restorations at 7 years. 16 MOD showing signs of marginal discolouration , wear and secondary caries.17 multi surface restoration still in good shape

Improvements in filler technology and the formulation of composite materials have resulted in changes in the reasons for restoration replacement, as well as the increasing trend to insert composite restorations in stress-bearing areas of posterior teeth [Figures 4-6].

The issue with restorative composites is to increase their flexure strength and fracture toughness, and thereby lengthen their service life in the oral cavity, while still maintaining their esthetic value. However, longevity and survival studies in posterior teeth continue to show that amalgam has a better track record than composite, further reinforcing the need to understand the failure mechanisms of dental composites.[19,20]

The recently developed resin composites are superior to the earlier versions, with regard to wear resistance and color stability, but the main shortcoming of the composites, i.e. the polymerization, shrinkage of the resin, still remains.[21–23] In posterior cavities, especially with the cervical margin situated in dentin, the mass to be polymerized is so large that the shrinkage forces win out, producing marginal defects and gaps, despite careful application.[24,25] This facilitates microleakage, which can cause secondary caries, pulpal irritation, postoperative sensitivity and marginal discoloration.[26,27]

## LONGEVITY OF COMPOSITE RESTORATIONS

Prospective clinical studies on posterior composite resin restorations show an annual failure rate of one to four percent, depending on the type of study and the materials selected.[15]

A 17-year study of ultraviolet-cured posterior composites by Wilder and others demonstrated an excellent success rate of 76%. Color matching (94% Alpha), marginal discoloration (100% Alpha), marginal integrity (100% Alpha), secondary caries (92% Alpha), surface texture (72% Alpha), anatomic form (22% Alpha) and a mean occlusal wear of 264 *µ*m were recorded after 17 years.

The improved reliability of composite resin as a restorative material in posterior teeth largely depends on the combination with an adhesive technique, which results in reduced microleakage and strengthening of weakened tooth structures.[28]

Hodge[29] found that the overall failure rate of the composite restorations in posterior teeth at eight years was 13.7% (16.4% for the microfilled composite; 15.4% for a fine particle hybrid composite; 9.3% for a relatively coarse particle hybrid composite). The failure rate of the composite restorations was approximately two to three times that of the control high copper amalgam restorations (5.8%). The main modes of failure of the composites were bulk fracture and secondary caries at the margin; these comprised 72% of the known modes of failures.

Raskin[30] found actual 10-year failure rate to have been between 40 and 50%. Cumulative approximal and related occlusal wear, with the resultant loss of contact areas, was found to be an important cause of failure, 10 years after placement.

However, any practical application of longevity data is somewhat offset by the fact that composite products are being modified or superseded almost constantly. The impact of advances in composite research and technology on the longevity of posterior restorations is evident in the retrospective study conducted by Baratieri and Ritter on the clinical performance of Class I and Class II composite restorations after four years. Although 2.5% of the restorations had clinically detectable marginal fracture, none required replacement.[31] Hickel *et al.*, in a meta-analysis, found the failure rate to be less than 9 %.[32]

The fact of the matter is that these materials are constantly improving to a point when new data is available; there is newer, stronger and improved material available.

The present ADA guidelines require an 18-month period of clinical service for acceptance of a new all-purpose composite resin. It might be better to have an evaluation period of at least five years before coming to any conclusions.

Interestingly, Opdam and colleagues, in a longitudinal study of over 700 posterior composite restorations placed by dental students, reported a 5-year survival rate of 87%, with an annual failure rate of 2.8%. They concluded that dental students are able to place resin composite restorations in posterior teeth, with an acceptable mean annual failure rate.[33]

## POLYMERISATION STRESSES

Shrinkage stresses have often been the bane of composite resin restorations. These stresses, which develop during polymerisation, lead to a host of issues including tooth flexure, post operative sensitivity and craze lines in marginal areas. Many factors affect the development of contraction stress in dental composites. These can be separated into material formulation factors (filler content, monomer chemistry, monomer structure, filler/matrix interactions, additives etc.) and material polymerization factors (polymerization rate, i.e. catalyst and inhibitor concentration, external constraint conditions, cavity geometry, curing method, placement technique etc.). Ferracane *et al.*,[34] in a systematic review, discussed the issue of polymerisation stresses in detail. They concluded that volumetric shrinkage should not be the only parameter to be considered for predicting composite behaviour regarding stress development. Materials with relatively low shrinkage, due to high inorganic filler content, also present high elastic modulus, which may result in increased stress.

Reduced polymerization rate, due to the use of alternative photo activation methods, does not necessarily lead to significant reductions in contraction stress. Also, a particular curing routine may not be efficient with composites from different manufacturers. Stress reduction cannot be achieved at the expense of adequate degree of conversion.[35]

Several new restorative techniques have been introduced during the last years, to minimize the development of stresses, such as multiple increment techniques, replacement of the dentin with glass ionomer cement in the sandwich technique.

## INDIRECT INLAYS

A promising method introduced to reduce the shrinkage problem was the resin composite inlay/onlay technique.[36,37] The form of the inlay can be established by either a direct or an indirect method. The clinical reports of heat-treated inlays do not confirm the suggested superior mechanical strength. It has been shown that the improvements of some of the properties were only short-time and these decreased due to weakening of the polymer by water uptake in the same way as for light-cured-only resin composites.[38]

Dijken,[39] in a 11-year evaluation of direct inlays and onlays, found good durability for the direct resin composite inlay/onlay technique. Excellent marginal adaptation and low frequency of secondary caries in patients with high caries risk were shown. No apparent improvement of mechanical properties was obtained by the secondary heat treatment of the inlays. Also, the difference in failure rate between the resin composite direct technique and the inlay technique was not large; indicating that the more time-consuming and expensive inlay technique may not be justified. The direct inlay/onlay technique is recommended to be used in Class II cavities of high caries risk patients, with cervical marginal placed in dentin. A review of inlay studies showed a low secondary caries rate in most of the evaluations.[40–41]

## THE SANDWICH TECHNIQUE

In the years when reliable dentin adhesive systems were not available, application of a glass-ionomer cement lining was the standard procedure to obtain bonding of the composite resin to dentin. Two variations of that type of restorations exist: the open and closed sandwich. In a closed sandwich, the dentin is covered with resin-modified glass-ionomer (RMGI) lining cement. In an open sandwich, RMGI is used to replace the dentin and also to fill the cervical part of the box, which results in a substantial part of the glass-ionomer cement being exposed to the oral environment.[42]

A lining with a low modulus of elasticity, such as a glass ionomer cement, is expected to act as a stress-absorbing layer and to compensate for polymerization shrinkage stress.[43]

Opdam,[42] in a retrospective study with a five-year observation period, made some interesting observations. Total-etch restorations placed with a highly filled hybrid composite resin showed a higher clinical survival than closed-sandwich restorations using a lining of RMGIC, due to a lower fracture rate. Fracture and secondary caries, the most important reasons for failure, mostly occurred after a period of more than three years.

It is often suggested that glass-ionomer cement should lead to a prevention of secondary caries. A lining of RMGIC is considered to act as an elastic layer, which should improve adaptation of the restoration and compensate for polymerization stress. Both these assumptions were disproved in this study, which questioned the alleged advantages of the elastic layer underneath a composite resin restoration.

Randall[44] found no data to support the cariostatic effects of GIC bases under restorations.

It would then be prudent to assume that the use of the RMGI liner should not be based on these criteria. The consensus, however, seems to suggest that increasing the compliance of the cavity walls by applying an intermediate low-modulus layer may lead to significant stress relief, depending on its thickness and elastic modulus. The alternative to RMGIC would be a flowable composite which would bond to dentine and provide a good seal.

Flowable composites may offer significant advantages when used as intermediate layers, according to the concept of radiopaque filled adhesives. They can also be used to improve adaptation to the cavity surface in areas that are difficult to access, especially when high-viscosity posterior composite materials are used subsequently.[45–46]

## POST OPERATIVE SENSITIVITY

Postoperative sensitivity is often mentioned in relation to posterior composite resin restorations. The introduction of self-etching primers, which do not remove the smear layer, has virtually eliminated the problem of postoperative sensitivity.[47] Two clinical studies that examined whether self etching adhesives result in less postoperative sensitivity than total-etch adhesives were not able to demonstrate a difference between the two methodologies.[48,49] Both studies found virtually no postoperative sensitivity with either technique. Present day adhesives, when used with care, following the manufacturer's instructions, produce little or no post operative sensitivity. Consistent post operative sensitivity could be attributed to faulty technique rather than a deficiency in the material.

Cavity size also exerts a significant effect in the survival of composite restorations. When compared to a one-surface restoration, the relative risk of failure is approximately 2.3 times greater for two-surface restorations and 3.3 times greater for multi-surface restorations. Reduction in cavity size will protect the restoration of the chewing forces.[15] Generally, multi-surface restorations will involve the marginal ridges (Class II), which are areas of increased loading. In a clinical trial of amalgam restorations, deterioration was greater in molars and large-sized restorations.[50] Another long-term (15 years) longitudinal study of amalgam restorations corroborates this study, showing that the replacement risk for MOD restorations was significantly higher than for MO/OD restorations.[51]

Additionally, Brunthaler et al.,[52] in a survey of prospective studies showed that filling extension influenced failure rates. (Class II fillings had higher failure rates.)

Van Nieuwenhuysen *et al.*,[53] in a prospective longitudinal study, evaluated extensive amalgam and composite restorations as substitutes for crowns. At the closure of the study, 48% of the restorations were well functioning, 24% were lost to lack of follow-up, and 28% had failed. The most frequent reasons for failure were fracture of restoration (8%), secondary caries (6%) and fracture of cusp (5%). Failures were more often found in premolar teeth (34%) than in molars (27%) and occurred in 28% of the amalgam restorations, 30% of the resin restorations and 24% of the crowns. Molar restorations were more frequently repaired than replaced, in contrast to premolar restorations. The highest percentage of extractions was related to complex amalgam restorations in premolars. The Kaplan-Meier median survival times were 12.8 years for amalgam restorations, 7.8 years for resin restorations, and more than 14.6 years for crowns, considering all retreatment as failures.

Rodolpho et al.[54] found that clinical performance of posterior resin composite restorations evaluated was acceptable after 17-year evaluation. These were restorations placed in a private practice and reflected clinical reliability of composites. They also found that the probability of failure of resin composite restorations in molars, Class II, and large restorations was higher. The materials used in the study, namely P 50 (3M) and Herculite XR (Kerr), have been surpassed by far superior materials.

**Figure 7:**
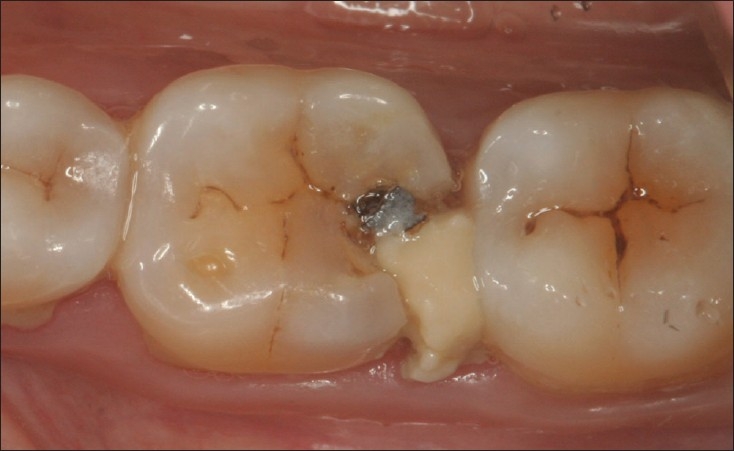
46 glass ionomer disto-occlusal restoration showing bulk fracture

**Figure 8:**
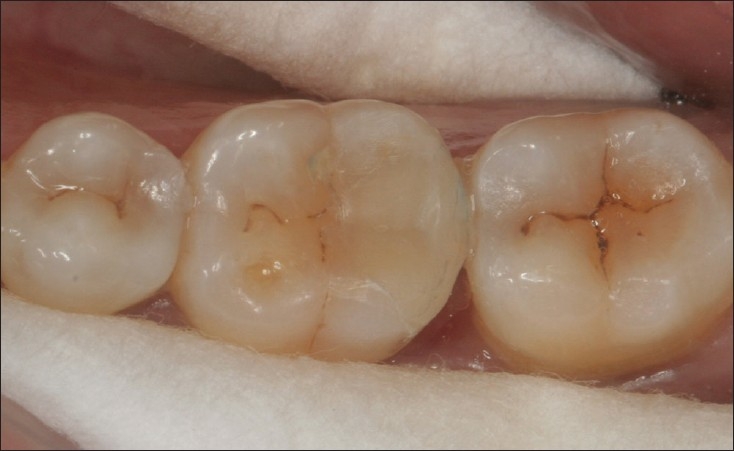
Fractured restoration replaced with Silorane based composite (P 90,3MESPE)

The most recent development in resin chemistry is based on using ring opening polymerization of the silorane molecules, instead of free radical polymerization of dimethacrylate monomers. Silorane resin reveals lower polymerization shrinkage, as compared to the dimethacrylates. The ring opening polymerisation of the silorane molecule is a cationic polymerization reaction, where no oxygen inhibition layer exists on the surface of the composite after polymerization in air.[55,56] Having had the opportunity to use this material, it holds promise for the future in eliminating the issue of shrinkage [Figures 7 and 8].

## COMPARATIVE EVALUATION OF LONGEVITY

Two recent studies with relatively long term follow ups provide partly contradictory results.

Bernardo *et al.*[20] evaluated a total of 1,748 restorations at baseline, which the authors followed for up to seven years. Overall, 10.1 percent of the baseline restorations failed. The survival rate of the amalgam restorations was 94.4 percent; that of composite restorations was 85.5 percent. Annual failure rates ranged from 0.16 to 2.83 percent for amalgam restorations and from 0.94 to 9.43 percent for composite restorations. Secondary caries was the main reason for failure in both materials. Risk of secondary caries was 3.5 times greater in the composite group.

Opdam *et al*,[57] found a survival rate of 91.7% for composite resin, at five years, and 82.2% at 10 years. For amalgam, the survival rate was 89.6% at five years and 79.2% at 10 years. Cox-regression analysis resulted in a significant effect of the amount of restored surfaces on the survival of the restorations. No significant effect of operator, material as well as combination of material and operator, was found.

One thing that is clear from these and other studies is that both amalgam and composite resins have excellent longevity, in excess of seven years. Interestingly, a unique and somewhat controversial study by Fairhurst *et al*.[58] provided some interesting insights. This 10-year study evaluated bonded and sealed composite restorations placed directly over frank cavitated lesions extending into dentin vs. sealed conservative amalgam restorations and conventional unsealed amalgam restorations. The results indicated that both types of sealed restorations exhibited superior clinical performance and longevity, when compared with unsealed amalgam restorations. Also, the bonded and sealed composite restorations placed over the frank cavitated lesions arrested the clinical progress of these lesions for 10 years.

## CONCLUSIONS

A random search of MEDLINE, limited to dental journals in the past five years, threw up some rather interesting results. Keywords such as ‘dental amalgam’ and ‘amalgam’ yielded 515 and 499 results respectively. The same search with the keyword ‘composite resin’ threw up 3271 results. A search on scholar.google.com yielded 28600 results for ‘dental amalgam’ and 361000 results for ‘composite resins’. This clearly reveals the shift away from amalgams towards composite resins. This shift has been brought about due to concerns about mercury toxicity as well as the general trend towards esthetic tooth coloured restorations. In clinical practice, most patients demand tooth coloured restorations and are reluctant to accept amalgams.

Based on this review, the following observations could be made:

1. Amalgam restorations have served the profession well and will continue to do so in the years to come. In terms of longevity, they are probably superior to composite resins, especially when used for large restorations and cusp capping. The newer high copper single composition alloys offer superior properties but may not offer as good a seal as older amalgams.
2. Composite resins are a viable alternative to amalgam for posterior restorations. They are more technique sensitive but offer a better seal and meet the patient's demands for esthetics. Fears about their longevity are unfounded and they perform well in clinical conditions. Their use in large restorations and in cusp capping situations is still a matter of debate.
3. In the years to come, dental students will be called upon to place composite restorations as soon as they graduate and enter clinical practice. Given the technique sensitivity and the learning curve associated with composites, it is time for our curriculum to absorb this change and provide more opportunities for students to hone their skills.
